# Segregation of a M404V mutation of the *p62/sequestosome 1 *(*p62/SQSTM1*) gene with polyostotic Paget's disease of bone in an Italian family

**DOI:** 10.1186/ar1828

**Published:** 2005-09-15

**Authors:** Alberto Falchetti, Marco Di Stefano, Francesca Marini, Francesca Del Monte, Alessia Gozzini, Laura Masi, Annalisa Tanini, Antonietta Amedei, Annamaria Carossino, Giancarlo Isaia, Maria Luisa Brandi

**Affiliations:** 1 Department of Internal Medicine, University of Florence, Florence, Italy; 2 Department of Internal Medicine, University of Turin, Turin, Italy; 3 DeGene Spin-off, University of Florence, Florence, Italy

## Abstract

Mutations of the *p62/Sequestosome 1 *gene (*p62*/*SQSTM1*) account for both sporadic and familial forms of Paget's disease of bone (PDB). We originally described a methionine→valine substitution at codon 404 (M404V) of exon 8, in the ubiquitin protein-binding domain of *p62*/*SQSTM1 *gene in an Italian PDB patient. The collection of data from the patient's pedigree provided evidence for a familial form of PDB. Extension of the genetic analysis to other relatives in this family demonstrated segregation of the M404V mutation with the polyostotic PDB phenotype and provided the identification of six asymptomatic gene carriers. DNA for mutational analysis of the exon 8 coding sequence was obtained from 22 subjects, 4 PDB patients and 18 clinically unaffected members. Of the five clinically ascertained affected members of the family, four possessed the M404V mutation and exhibited the polyostotic form of PDB, except one patient with a single X-ray-assessed skeletal localization and one with a polyostotic disease who had died several years before the DNA analysis. By both reconstitution and mutational analysis of the pedigree, six unaffected subjects were shown to bear the M404V mutation, representing potential asymptomatic gene carriers whose circulating levels of alkaline phosphatase were recently assessed as still within the normal range. Taken together, these results support a genotype–phenotype correlation between the M404V mutation in the *p62*/*SQSTM1 *gene and a polyostotic form of PDB in this family. The high penetrance of the PDB trait in this family together with the study of the asymptomatic gene carriers will allow us to confirm the proposed genotype–phenotype correlation and to evaluate the potential use of mutational analysis of the *p62*/*SQSTM1 *gene in the early detection of relatives at risk for PDB.

## Introduction

Paget's disease of bone (PDB; Online Mendelian Inheritance in Man (OMIM) entry no. 602080) is a metabolic bone disease characterized by accelerated bone resorption followed by the deposition of dense, chaotic bone matrix, affecting up to 3% of individuals of Caucasian ancestry above the age of 55 years [1]. Although PDB is genetically heterogeneous, in some familial cases of late onset PDB an autosomal dominant pattern of inheritance has been reported [2-4]. Mutations of the *p62*/*sequestosome 1 *(*p62*/*SQSTM1*) gene account for most of the sporadic and familial forms of PDB [1-5], and exons 7 and 8, encoding the ubiquitin-binding-associated domain (UBA), host a clustered mutational area [2-5]. p62 acts as a scaffold protein in signalling pathways downstream of the interleukin-1, tumour necrosis factor (TNF)-α and nerve growth factor receptors [6].

In a recent paper we described an M404V mutation in the UBA of the *p62*/*SQSTM1 *gene in an Italian population of patients affected by PDB [5]. This mutation has also been confirmed in other ethnic groups [7-9]. For the Italian patient carrying this A→G transition at exon 8 [5], collection of the family history demonstrated a clear inheritance for PDB. DNA analysis for the *p62*/*SQSTM1 *gene mutation was performed in all affected familial members and in several unaffected subjects, to evaluate the segregation of the M404V mutation with the PDB phenotype and to detect potentially asymptomatic gene carriers. Through this analysis we identified both a familial form of PDB, in which the M404V mutation segregates with a polyostotic phenotype of the disorder, and several asymptomatic gene carriers.

## Materials and methods

### Family recruitment and disease ascertainment

**Figure 1:**
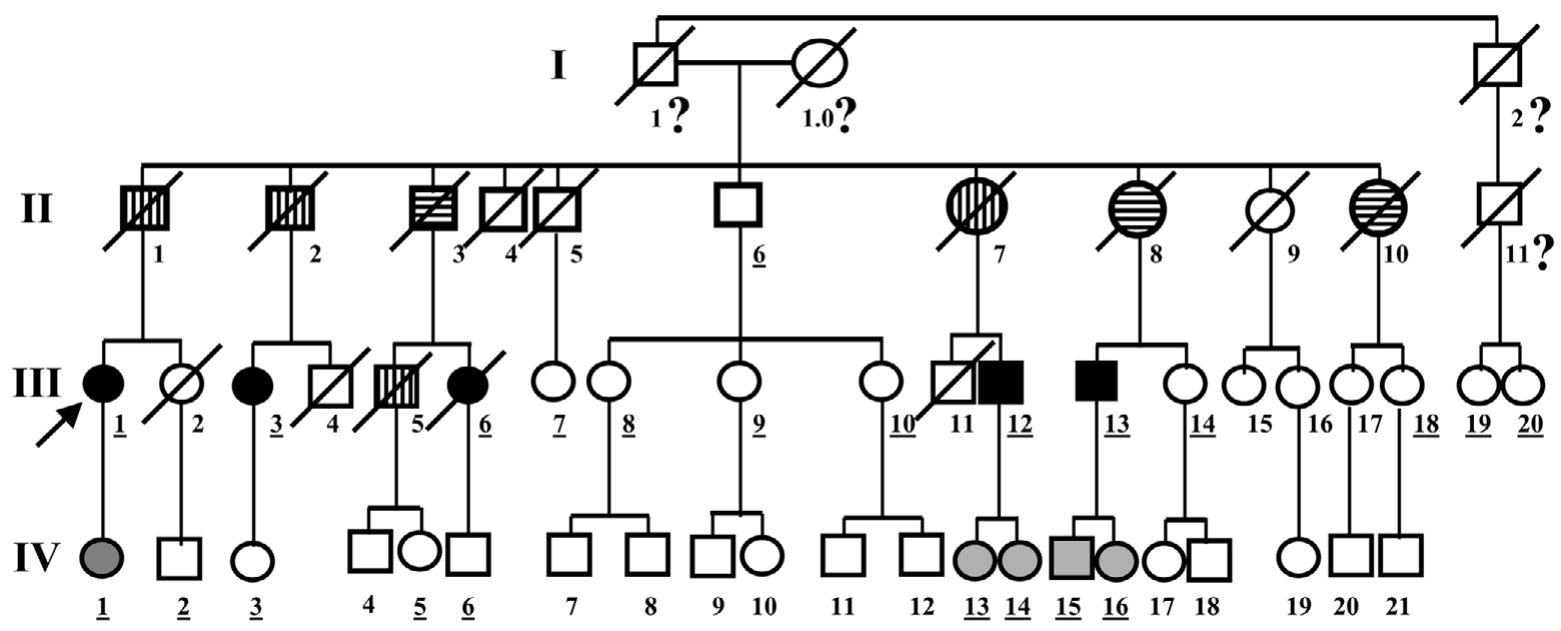
Family pedigree (F01). The proband, III-1, is indicated by an arrow. Members of family with ascertained clinical evidence of Paget's disease of bone (PDB; III-1, III-3, III-6, III-12 and III-13) are represented by black symbols. Subjects strongly suspected to be affected by PDB, as reported by personal history in relatives (II-3, II-8 and II-10), are indicated by horizontal bar symbols. Relatives potentially mutant on the basis of pedigree reconstruction (II-1, II-2, II-7 and III-5) are represented by vertical bar symbols. Grey symbols identify individuals known to have the M404V mutation but whose PDB disease was not expressed; open symbols indicate subjects not exhibiting either the mutation or clinical evidence of PDB. Underlined numbers indicate individuals in whom a genetic test was performed. Question marks identify individuals whose clinical phenotype is not verifiable.

The Local Ethical Committee of the University of Florence approved this study. The PDB female proband (III-1) was clinically evaluated and genetically characterized as a carrier of a novel M404V mutation at exon 8 of the *p62*/*SQSTM1 *gene (Fig. 1) [5].

Through the family history a familial form of PDB (F01 pedigree, Fig. 1) was ascertained. The four-generation family, originating from central Italy, consists of 37 living subjects (22 females and 15 males; age range 33 to 92 years) and 18 deceased individuals (11 males and 7 females; Fig. 1). Members from generations I to III were farmers born and still living in a rural environment, whereas fourth-generation individuals, although born in the same environment as previous generations, moved to urban life after adolescence. Relevant clinical information on affected and gene-carrier members of the F01 pedigree was collected; they are summarized in Table 1.

All available family members were asked to undergo DNA mutational analysis and biochemical assessment after administration of an informed consent form.

No information was available on the first (I) generation (subjects I-1, I-1.0 and I-2; Fig. 1).

**Figure 2:**
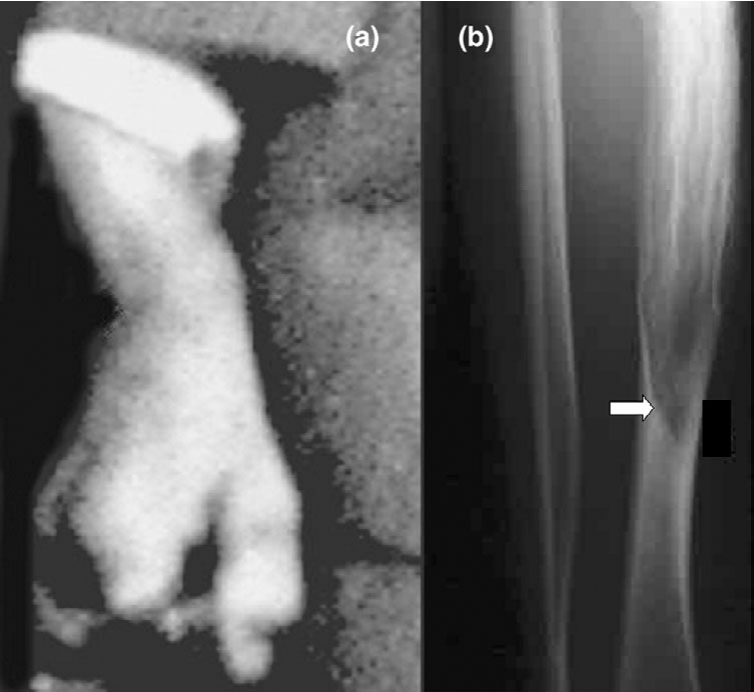
Evidence of PDB in two affected subjects from F01 family. **(a) **Bone deformity of the right forearm of family member II-3. Relatives described him as having suffered from multiple bone deformities and pain. **(b) **X-ray scan of the right tibia of family member III-3, with a typical flame-shaped lytic wedge (arrow).

In the second (II) generation (Fig. 1) blood samples for genomic DNA evaluation were obtained from the only living subject (patient II-6), a 92-year-old male, suffering from a benign hyperplasia of the prostate (Table 1). Male subject II-3, the father of a III-6 affected individual (Fig. 1), was referred to as a carrier of multiple bone deformities and pain by living members of the family, strongly suggesting the presence of PDB disease in this individual. Figure 2a contributes to sustaining this hypothesis.

The proband III-1, in whom the M404V mutation was first detected [5], belongs to the third (III) generation (Fig. 1). Patient III-6 died because of an osteogenic sarcoma within or in a Pagetic bone. This patient was diagnosed as being affected by PDB at the age of 62 years because of the presence of bone pain, elevated serum alkaline phosphatase (AP) activity and X-rays indicating typical PDB. Bone scintigraphy showed signs of disease in the right pelvis, the right proximal femur and left ribs IV and VIII. Three years after the diagnosis of PDB, bone pain in the right pelvis increased markedly and a bone biopsy showed the presence of an osteogenic sarcoma on the Pagetic bone already metastasized to the lungs. The patient died at 65 years of age after surgical and chemotherapeutic interventions, some years before DNA analysis was performed on the F01 pedigree.

The ages of members from the fourth (IV) generation (IV-1, IV-2, IV-3, IV-5, IV-6, IV-13, IV-14, IV-15 and IV-16) ranged from 41 to 53 years. Neither clinical nor biochemical abnormalities suggestive of PDB are currently evident in this younger group (Fig. 1, Table 1).

Evaluation of AP, measured by an autoanalyzer, has been performed also in all the individuals undergoing mutational analysis. The upper limit of the reference range is 120 units/l.

### DNA extraction, PCR and mutational analysis

After administration of an informed consent form, peripheral blood was obtained from 22 subjects: 4 PDB patients (III-1, III-3, III-12 and III-13) and 18 clinically unaffected members (II-6, III-7, III-8, III-9, III-10, III-14, III-18, III-19, III-20, IV-1, IV-2, IV-3, IV-5, IV-6, IV-13, IV-14, IV-15 and IV-16) (Fig. 1, Table 1). Genomic DNA was extracted from peripheral blood leukocytes with the use of a microvolume extraction method, QIAamp DNA Mini Kit (Qiagen GmbH, Hilden, Germany), in accordance with the manufacturer's instructions.

Exon 8 of the *p62*/*SQSTM1 *gene was amplified by PCR (I-Cycler; Bio-Rad Laboratories, Milan, Italy) using a couple of primers located in the flanking intron: 5'-CAGTGTGGCCTGTGAGGAC-3'/5'-CAGTGAGCCTTGGGTCTCG-3'. For each patient we used 0.1 μg of DNA, in a final buffer volume of 50 μl (67 mM Tris-HCl, 16.6 mM (NH_4_)SO_4_, 0.01% Tween 20, 1.5 mM MgCl_2_, 0.2 mM deoxyribonucleotides, each primer at 0.2 μM and 1 unit of Polytaq (Polymed, Florence, Italy)). Thirty PCR cycles were performed at 94°C for 30 s, 55°C for 30 s and 72°C for 1 min, after a first denaturing cycle at 94°C for 3 min. A final extension cycle of 5 min was performed at 72°C.

PCR products were tested by 2% ethidium bromide-stained agarose-gel electrophoresis, purified with a High Pure PCR Product Purification Kit (Roche, Indianapolis, IN, USA) and finally sequenced with a BigDye Terminator v3.1 Cycle Sequencing Kit (Applied Biosystems, Foster City, CA, USA). The sequencing reaction consisted of 25 repeated cycles of denaturation for 10 s at 96°C, annealing for 5 s at 55°C and extension for 2 min at 60°C. The sequencing products were purified with a DyeEx 2.0 Spin Kit (Qiagen GmbH, Hilden, Germany) to remove the excess dye terminator. A 5 μl sample of each purified sequence was then resuspended in 15 μl of formamide and denatured for 2 min at 95°C. Analysis of the forward and reverse sequences was performed on an ABI Prism 3100 Genetic Analyzer (Applied Biosystems, Foster City, CA, USA).

## Results

### Clinical data

Suspicion or diagnosis of PDB was based on description by relatives, evidence of bone deformities and pain strongly suggestive of PDB (II-3 (Fig. 2a), II-8 and II-10) and, when possible, direct evidence of elevated total AP, X-ray scanning and bone scintigraphy (III-1, III-3 (Fig. 2b), III-6, III-12 and III-13; Fig. 1, Table 1).

After careful reconstitution of their clinical history, 12 subjects (6 males and 6 females), of which 11 were living, were reported to be the following: clinically ascertained as PDB patients (III-1, III-3 (Fig. 2b), III-6, III-12 and III-13) with increased circulating levels of AP (more than 120 units/l); presumably affected by PDB (II-3 (Fig. 2a), II-8 and II-10); and potentially mutant (II-1, II-2, II-7 and III-5; Table 1).

In accordance with previously described criteria, all affected members, clinically ascertained (III-1, III-3 (Fig. 2b), III-6, III-12 and III-13), exhibited polyostotic localization of PDB (III-1, III-3, III-6 and III-13), except patient III-12 (Table 1). In the last of these the monostotic left femur involvement was diagnosed only through standard X-ray examination, so the possibility of underestimation of skeletal involvement cannot be excluded (Table 1). Over all, considering only the clinically ascertained affected subjects (III-1, III-3, III-6, III-12 and III-13), the number of bones involved in this family is 3.8 ± 2.31 (mean ± SD). Three subjects in the family (II-3 (Fig. 2a), II-8 and II-10) were presumably affected by PDB on the basis of the history related by other members of the family and of the fact that progeny of II-3 and II-8 carried the M404V mutation. In these three cases the description of bone deformities was suggestive of multiple bone localization.

Until now none of the unaffected members has exhibited AP levels outside the normal range (that is, more than 120 units/l).

### Mutational analysis

All the available ascertained affected PDB individuals from the third generation (III-1, III-3, III-12 and III-13) exhibited the M404V mutation of the *p62*/*SQSTM1 *gene (Table 1), confirming the pathogenetic nature of this *p62*/*SQSTM1 *gene mutation and suggesting segregation of the mutation with the polyostotic phenotype in this family. Although patient III-6 died as a result of an osteogenic sarcoma on a Pagetic bone several years before the genetic evaluation of F01 pedigree, he probably had the M404V mutation. Through mutational analysis the pedigree was carefully reconstructed, and this allowed us to propose that patients II-1, II-2, II-3, II-7, II-8 and III-5 were also carriers of the M404V mutation. In fact, their PDB-affected children (III-1, III-3, III-6, III-12 and III-13) exhibited the mutation (Table 1).

Of the unaffected subjects, II-6, III-7, III-8, III-9, III-10, III-14, III-18, III-19, III-20, IV-2, IV-3, IV-5 and IV-6 (age range from 35 to 92 years) were not carrying the M404V *p62*/*SQSTM1 *gene mutation, whereas subjects IV-1, IV-13, IV-14, IV-15 and IV-16 (age range from 41 to 53 years) were carrying the mutation (Table 1).

AP levels were still in the normal range (less than 120 units/l) in these gene carriers (Table 1).

## Discussion

Several lines of evidence [2-7] support the role of the *p62*/*SQSTM1 *gene in the pathogenesis of PDB, even though the molecular mechanisms that underlie its functional activities are not fully understood. Similarly, little information has been collected about either the potential genotype–phenotype correlation between gene mutations and clinical manifestations of PDB [7] or the role of genetic testing in asymptomatic carriers within affected families. The findings described in this paper are of interest with regard to both issues.

The p62/SQSTM1 protein binds non-covalently to ubiquitin, co-localizing with ubiquitinated inclusions in several human diseases characterized by altered protein aggregation [10]. Moreover, the protein mediates several cellular functions including NFκB-dependent signalling and transcriptional activity, which are important for the recruitment and activation of osteoclastic cells [2].

The nuclear magnetic resonance structure of the p62-UBA domain has recently been determined, but its functional significance in the p62 protein is still unknown [11]. The study by Ciani and colleagues showed that the M404V mutation is able to modify the secondary structure of the domain and affects its ability to bind to Lys^48^-linked multiubiquitin chains *in vitro *[11].

Together with other *p62*/*SQSTM1 *gene mutations at the UBA domain, Cavey and colleagues [12] showed that M404V is able to cause the loss of monoubiquitin binding and impair *in vitro *Lys^48^-linked polyubiquitin binding, although these effects were reported only when the binding experiments were performed at the physiological temperature of 37°C. These findings suggest that PDB-related *SQSTM1 *mutations may confer a higher susceptibility to development of the disease by impairing the binding of the p62 protein to a ubiquitinated target. However, other molecular mechanisms, involving a key ubiquitinated substrate, could be invoked in the attempt to explain the acquisition of the PDB phenotype in individuals with mutations of the *p62*/*SQSTM1 *gene [11]. A structural analysis demonstrated in 4 of 70 PDB relatives with British ancestry that an M404V mutation involves residues on the hydrophobic surface patch implicated in ubiquitin binding [7]. Consequently, an M404V mutation affects the ability of a mutant UBA domain to bind polyubiquitin chains [7].

Using this structural information Hocking and colleagues reported that patients with truncating mutations of the *p62*/*SQSTM1 *gene exhibited a trend for more extensive PDB than those with mis-sense mutations such as M404V. They concluded that there is no correlation between the ubiquitin-binding properties of different mutant UBA domains and disease occurrence or extension of the same [7]. These findings therefore do not provide a speculative hypothesis for an explanation of the observed genotype–phenotype correlation in the Italian family with PDB described in this paper.

The heterozygous segregation of M404V mutation with the PDB phenotype in the F01 pedigree supports the pathogenetic role via a dominant-negative action [4]. Moreover, the evidence of a genotype–phenotype correlation in this family can also include epigenetic mechanisms that, through a common genetic background, can contribute, along with the M404V mutation, to the expression of a polyostotic PDB phenotype in the affected members. Interestingly, the commonly shared rural environment of all the members from generations I to III and, for a shorter period, generation IV of this family, together with the presence of past measles infection in all individuals analysed, could suggest a role for environmental factors in determining the polyostotic expression of the disease in M404V mutant subjects.

Clinical follow-up of asymptomatic carriers from generation IV might confirm or negate this observation. Studies on the penetrance of PDB have been performed by several authors [2-4,9], the PDB shows an incomplete clinical expression, meaning that some *SQSTM1 *gene carriers from affected families do not show clinical evidence of the disease. Moreover, some PDB-affected individuals, from affected families with a known *SQSTM1 *gene mutation, do not exhibit the mutation [2-4,9]. Conversely, individuals older than 55 years of age with a known *SQSTM1 *mutation from relatives affected with PDB, did not develop PDB [4]. A potential explanation for these findings is the existence of genetic heterogeneity, with possible modifier loci capable of controlling the clinical expression of PDB [4,9]. For individuals younger than 55 years of age with a known *SQSTM1 *mutation, originating from relatives affected with PDB, who had not yet developed PDB [4,8], the time needed for phenotypic expression of the disease could represent a limiting factor. In general, a lack of expression of the disease in recognized *SQSTM1 *gene carriers could be explained by a reduced exposure to environmental factors such as paramixovirus infections and/or by the progressive abandonment of the rural environment [4,7].

An important application of genetic analyses in families is the precocious identification of asymptomatic gene carriers. In this relative the *p62*/*SQSTM1 *disease-associated mutation was also present in individuals younger than 50 years of age [3,4]. So far the asymptomatic gene carriers have not exhibited any abnormality in the circulating levels of AP and have not shown any clinical signs suggestive of PDB. Although bone scanning is commonly recommended in patients with PDB older than 40 years of age, because of the ethical considerations observed in our country, bone scan tests cannot be performed unless AP levels are raised and consequently PDB bone localization cannot be excluded at this stage in asymptomatic mutant carriers. However, considering that a positive individual older than 40 years of age has an up to 80% likelihood of developing the disease by 70 years of age [13], the extremely high penetrance of PDB in this family clearly indicates the need for an accurate vertical follow-up of the six asymptomatic mutant carriers. This will allow us to confirm the suggested genotype–phenotype correlation in the currently asymptomatic carriers as well, and to assess the role of mutational analysis of the *p62*/*SQSTM1 *gene for early detection of the individuals at risk for developing PDB. At present, a positive test for the mutation of the *p62*/*SQSTM1 *gene in a patient with PDB does not have any impact on treatment [13].

Finally, one of the affected subjects (III-6) in this family developed an osteosarcoma that caused her death. Pagetoid osteosarcoma is a complication of PDB [14,15] and is most often observed in severe, long-standing PDB. Two previous reports described a direct lineage in which Pagetoid osteosarcoma developed in affected family members [16,17]. Although specific genetic mechanisms remain to be elucidated, some authors reported loss of heterozygosity for loci at chromosome 18q21-22 in Pagetoid osteosarcomas as well as in sporadic osteosarcomas [17,18]. The deleted region was shown to harbour the receptor activator of nuclear factor κB (*RANK*, *TNFRSF11A*) gene identified in a family affected by familial expansile osteolysis (OMIM entry no. 174810), a Paget-like syndrome [19]. Although the *RANK *gene has not been found to be mutated in PDB-affected individuals, a positive association between a polymorphic variant of this gene and PDB has been reported [20,21]. NFκB is also the potential molecular target of the mechanism underlying the altered osteoclastogenesis seen in PDB patients carrying mutated sequences in the UBA domain of the *p62*/*SQSTM1 *gene [2-12]. Inactivation of the *p62*/*SQSTM1 *gene could activate the RANK–NFκB signalling, as seen in the familial expansile osteolysis syndrome [19], with impairment of TNF-α-induced programmed cell death [22]. Such machinery is also crucial for immunity, lymphocyte development, tumorigenesis and cancer chemoresistance; NFκB functions are recognized as relevant to tumour promotion [23]. Even though the presence of the M404V mutation could not be assessed in patient III-6, these hypotheses strongly support the need for further investigation into the possible role of the p62 protein in the occurrence of osteosarcoma, both in PDB-affected patients and in individuals without PDB.

## Conclusion

This paper describes a genotype–phenotype correlation in PDB cases with a mis-sense mutation in the *p62*/*SQSTM1 *gene. These results should be confirmed in other PDB patients of Italian and other ancestries. Moreover, the value of a pre-symptomatic gene test in PDB requires a vertical evaluation in well-characterized relatives, opening new possibilities for the practical application of genetic diagnosis in PDB family members and also in the general population. Finally, the knowledge of the function of *p62*/*SQSTM1 *gene mutations should enable us to uncover the pathogenesis of PDB and osteogenic osteosarcoma.

## Abbreviations

AP = alkaline phosphatase; NFκB = nuclear factor κB; PCR = polymerase chain reaction; PDB = Paget's disease of bone; RANK = receptor activator of nuclear factor κB; TNF = tumour necrosis factor; UBA domain = ubiquitin-binding-associated domain.

## Competing interests

The author(s) declare that they have no competing interests.

## Authors' contributions

AF conceived of the study and participated in its design and coordination, acquisition of data, analysis and interpretation of data, and was fully involved in drafting the manuscript and revising it critically for important intellectual content. MDS made substantial contributions to the acquisition and interpretation of data. AF and MDS contributed equally to the work. FM performed the molecular genetic studies. FDM performed the molecular genetic studies together with FM. AG participated in the sequence alignment. LM performed the statistical analysis. AT supervised the performance statistical analysis. AA helped in the clinical activity. AC participated in the sequence alignment. GI participated in the design of the study and helped to draft the manuscript. MLB participated in the design and coordination and helped in drafting the manuscript and revising it critically for important intellectual content; she also gave final approval of the version to be published. All authors read and approved the final manuscript.

## Figures and Tables

**Table 1:** Available clinical and mutational data on affected patients and gene carriers of PDB

Pedigree number (sex)	Age at clinical diagnosis or DNA evaluation; present age (years)	AP (U/l)	PDB-related clinical finding	Other relevant clinical data
II-3 (M)^a^	Deceased	Unknown	Diffuse marked bone deformities (Fig. 2a)	Died at age 76 years from Alzheimer's disease
II-8 (F)^a^	Deceased	Unknown	Bone deformities at both lower extremities	Died at age 52 years from colon-rectal cancer; also had breast cancer
II-10 (F)^a^	Deceased	Unknown	Multiple marked diffuse skeletal deformities	Died at age 92 years from unknown cause
**III-1 (F)**^b^	57; 65	357	Third lumbar vertebra, pelvis, right proximal femur	Alive
**III-3 (F)**^b^	53; 79	560	Pelvis, both tibias	Alive. Apparently healthy
III-5 (M)^a^	Not assessed	Unknown	Diffuse bone pain	Died at age 80 years from unknown cause
III-6 (F)^a^	62; deceased	2,259	Right pelvis and proximal femur, IV and VIII left ribs	Died 12 years previously from osteogenic sarcoma on Pagetic bone (right pelvis)
**III-12 (M)**^b^	64; 82	380	Left hip^c^	Alive. Benign prostate hyperplasia
**III-13 (M)**^b^	65; 83	610	T5, T10 and L4 vertebral bodies, sacrum, right tibia, right femur, right shoulder and collarbone	Alive. Allergy to pollen, hypertensive cardiopathy
**IV-1 (F)**	41	<120	None	Alive, age 42, healthy
**IV-13 (F)**	53	<120	None	Alive, hypertension, age 54
**IV-14 (F)**	41	<120	None	Alive, age 42, lumbar–sacral discal hernia, goitre
**IV-15 (M)**	47	<120	None	Alive, age 48 allergy to pollen
**IV-16 (F)**	48	<120	None	Alive, age 49, allergy to pollen

The M404V mutation was ascertained in individuals listed in bold. The highest observed levels of alkaline phosphatase (AP) are reported for each affected subject; the normal range is less than 120 units/l. PDB, Paget's disease of bone.

^a^Individuals strongly suspected to be potential PDB patients after careful reconstruction of the familial clinical history. ^b^These subjects received two treatment courses with oral risedronate (30 mg/day) for 3 months followed by a 112-day follow-up period without treatment [24]; complete normalization of serum AP levels and bone pain remission were observed in all these treated subjects. ^c^Total bone scintigraphy was not performed on this subject; the skeletal extent of PDB is on the basis of X-ray evaluations.
